# Comparison of efficacy and safety of complementary and alternative therapies for scapulohumeral periarthritis: A protocol for Bayesian network meta-analysis

**DOI:** 10.1097/MD.0000000000025769

**Published:** 2021-05-07

**Authors:** Meihan Gao, Haibo Cong, Chuancheng Li, Xiuyun Qin, Hongqiang An, Zhenyuan Jiang

**Affiliations:** a The First Clinical College, Shandong University of Traditional Chinese Medicine; b Weihai Central Hospital, China.

**Keywords:** complementary and alternative therapies, protocol., scapulohumeral periarthritis, web meta-analysis

## Abstract

**Background:**

Scapulohumeral periarthritis is a disease with high incidence and great pain. The current western treatments with many side effects, poor efficacy cannot fundamentally solve the problem. Complementary and alternative therapies have played an excellent role in the treatment of scapulohumeral periarthritis. However, it is not clear which complementary and alternative therapy is more effective. Therefore, we propose a protocol to compare the efficacy and safety of various complementary and alternative therapies through network meta-analysis (NMA) to provide choice guidance for the therapy.

**Methods:**

A comprehensive search will be conducted for randomized controlled trials of complementary and alternative therapy for scapulohumeral periarthritis as well as ongoing trials. The time limit is from the establishment of the database until January 2021. Literature and data extraction were completed independently by two researchers. Through pairwise comparison and meta-analysis of Bayesian NMA, all the evidences are evaluated comprehensively. STATA16.0 and WinBUGS1.4.3 software will be used for data processing and analysis, and recommendation evaluation will be used to develop and assess grades to classify the quality of NMA evidence.

**Results:**

Through the analysis, we will obtain the ranking of the efficacy and safety of different complementary and alternative therapies in the treatment of scapulohumeral periarthritis, in order to provide reference for clinical selection of treatment methods.

**Conclusion:**

Complementary and alternative therapies of scapulohumeral periarthritis plays a positive role in improving the symptoms of scapulohumeral periarthritis. This study can provide evidence support for clinicians and patients.

International Platform of Registered Systematic Review and Meta-Analysis Protocols registration number: INPLASY202140044.

## 1 Introduction

Scapulohumeral periarthritis (SP) is a disease with chronic and specific inflammatory change caused by degeneration and strain of muscle, ligament, tendon, synovial capsule and joint capsule around shoulder joint. The incidence of scapulohumeral periarthritis is 2% to 5%,^[1–4]^ accounting for about 42% of shoulder diseases. It is more common in the middle-aged and elderly and has a younger trend at present. The incidence in women is higher than that in men.^[1,5]^ Its main manifestations are shoulder pain and limitation of shoulder movement. The initial pain is paroxysmal. With the progress of the disease, it can be transformed into persistent and severe pain, usually aggravated at night, which can seriously affect sleep.^[3–6]^ It is often accompanied by limitation of movement,^[3,8]^ even accompanied by different degrees of deltoid muscle atrophy.^[9]^

Scapulohumeral periarthritis is a chronic disease with a long course, severe shoulder pain, limited movement and a high disability rate, which makes patients suffer extreme pain, prone to insomnia, anxiety and other psychological problems, seriously affecting the quality of life.^[10–12]^ Therefore, there is an urgent need to find a way to treat the disease. At present, the main methods of western medicine in the treatment of SP are oral drugs, local anesthesia, local blocking therapy, physiotherapy, surgical treatment, etc.^[4]^ Surgical treatment has limited indications, high cost and more risks.^[13]^ Although there are many other treatments, which can temporarily relieve symptoms, the long-term effect is not good,^[14]^ and western oral medicine has certain side effects.

Complementary and alternative therapies for SP consist of many methods, such as Chinese Herbal medicine, acupuncture, moxibustion, massage, exercise, yoga, Baduanjin, Taichi, and cupping therapy. They possess advantages of sound and lasting therapeutic effects and few side effects, etc. Nevertheless, reviewing all the current researches, we discovered that there's a lack of systematic analysis of the efficacy and safety of various complementary and alternative therapies which can rank their efficacy. Based on these findings, we conducted a study of complementary and alternative therapies in the treatment of scapulohumeral periarthritis, and proposed a network meta-analysis (NMA) protocol to explore the efficacy of different complementary and alternative therapies.

## 2 Methods

In this study, we will use NMA, and conduct a literature review in accordance with the PRISMA statement.^[15]^

### 2.1 Study registration

This NMA has been registered on the International Platform of Registered Systematic Review and Meta-Analysis Protocols (INPLASY). The registration number is INPLASY202140044 (URL: https://inplasy.com/inplasy-2021-4-0044/).

### 2.2 Inclusion criteria

#### 2.2.1 Type of study

We will include (RCT) randomized controlled trials of complementary and alternative therapies related to this study published in China and internationally. The language is limited to Chinese and English.

#### 2.2.2 Participants

In accordance with internationally recognized diagnostic criteria, patients diagnosed as SP will be included, regardless of source, sex, age, etiology, course, severity, and race. And there must be clear criteria for evaluating therapeutic effects.

Interventions and comparators: The experimental group was treated with Chinese Herbal Medicine, acupuncture, moxibustion, massage, exercise, yoga, Baduanjin, Taichi, and cupping therapy. All the above kinds of treatments can be used alone or in combination. The control group was given routine treatments such as western medicine, placebo, non-treatment, etc.

#### 2.2.3 Results

Main results: VAS score (visual analogue scale), Shoulder motor function score, effective rate.

Secondary results: PGE2, 5-HT, inflammatory indexes TNF- α, IL-6, adverse reactions, and other indicators.

### 2.3 Exclusion Criteria

Animal experiments and other studies, non-randomized controlled trials, studies that do not have clear criteria for therapeutic effects evaluation, reviews, poorly designed studies, cross-sectional studies, repetitive or plagiarized articles.

### 2.4 Search strategy

We will search PubMed, CNKI, Wanfang database, CochraneLibrary, VIP database (VIP), EMBASE, Web of Science, and Cochrane Central Register of Control Trials, Clinical Trials.gov clinical registration system. The language is limited to Chinese and English. The time range of retrieval is from the date of the establishment of the database to January 30, 2021. The retrieval skills and attentions will be studied in detail, and the retrieval will be carried out by a combination of subject words and free words. The final retrieval strategy is determined after many searches. We will collect all completed or ongoing RCT of complementary and alternative therapies for SP.

Take PubMed as an example, the retrieval strategy is shown in Table 1.

**Table 1:** Search strategy for Pubmed.

NO.	Search item
#1	“periarthritis of shoulder”[MeSH Terms] OR“scapulohumeral periarthritis”[MeSH Terms] OR “adhesive periarthritis of shoulder”[MeSH Terms] OR “frozen shoulder”[MeSH Terms] OR “Shoulder Pain”[Mesh]
#2	“scapulohumeral periarthritis”[Title/Abstract] OR “Frozen Shoulders”[Title/Abstract] OR “Shoulder, Frozen”[Title/Abstract] OR “Adhesive Capsulitis of the Shoulder”[Title/Abstract] OR “Shoulder Adhesive Capsulitis”[Title/Abstract] OR “Adhesive Capsulitis, Shoulder”[Title/Abstract] OR “Capsulitis, Shoulder Adhesive”[Title/Abstract] OR “Adhesive Capsulitis”[Title/Abstract] OR “Capsulitis, Adhesive”[Title/Abstract] OR “stiff shoulder”[Title/Abstract] OR “Shoulder osteoarthritis”[Title/Abstract] OR “Shoulder osteodystrophy”[Title/Abstract]
#3	#1 OR #2
#4	“Complementary Therapies”[Mesh Terms]
#5	“alternative therapies”[Title/Abstract] OR “Therapies, Complementary”[Title/Abstract] OR “Therapy, Complementary”[Title/Abstract] OR “Complementary Medicine”[Title/Abstract] OR “Medicine, Complementary”[Title/Abstract] OR “Alternative Medicine”[Title/Abstract] OR “Medicine, Alternative”[Title/Abstract] OR “Alternative Therapies”[Title/Abstract] OR “Therapies, Alternative”[Title/Abstract] OR “Therapy, Alternative”[Title/Abstract]
#6	#4 OR #5
#7	“acupuncture”[Title/Abstract] OR “cupping therapy”[Title/Abstract] OR “moxibustion”[Title/Abstract] OR “massage”[Title/Abstract] OR “yoga”[Title/Abstract] OR “taichi”[Title/Abstract] OR “Chinese herbal medicines”[Title/Abstract] OR “Baduanjin”[Title/Abstract] OR “exercise therapy”[Title/Abstract]
#8	#6 OR #7
#9	“Randomized Controlled”[Publication Type] OR “Controlled Clinical Trial”[Publication Type] OR “Randomized”[Title/Abstract] OR “Randomly”[Title/Abstract] OR “random allocation”[Title/Abstract]
#10	#3 AND #8 AND #9

### 2.5 Research selection and data extraction

In this study, we will use EndNoteX9 for detection. According to the predetermined retrieval strategy, all the researches retrieved in the above database and manually retrieved are imported into EndNoteX9 for classification and management. Two researchers independently screened the literature, extracted the data, and recorded the data in the MicrosoftExcel2019 software at the same time. If there is a difference of opinion, the two researchers will make a decision after discussion, with the participation of a third researcher if necessary.

The following data will be recorded: title, country, journal, first author, publication time, sample size, race, diagnosis, age, case source, course of disease, inclusion criteria, exclusion criteria, intervention measures, process, main research indicators, and results. If the relevant data is incomplete, we will try to contact the author to obtain the relevant data information. If the relevant data is still not available, we will only analyze the available data and explain the possible impact of the missing data.

### 2.6 Risk of bias assessment

In this study, the deviation risk assessment tool recommended in the Cochrane system reviewer's Handbook 5.3 will be used to evaluate the quality of the included literature. It was carried out independently by two researchers (GMH and LCC). If there is a disagreement, the decision will be made through discussion. When necessary, the third researcher (QXY or JZY) will make the decision and explain the reason. The evaluation criteria will include the following aspects: correct application of randomization, the application of allocation hiding, the blindness of participants and researchers, the integrity of results and data, selective reporting of results, and other related biases. According to the above criteria, the bias risk included in the study is divided into three levels: “low bias risk,” “high bias risk” and “ambiguous bias risk.”

### 2.7 Statistical analysis

We will use Stata16.0 software for paired Meta analysis and WinBUGS software for Bayesian NMA statistical analysis. Odds ratio will be adopted for counting data, Mean difference (MD) or standardized mean difference (SMD) will be adopted for continuous variable data. Therapeutic effects are evaluated by the effect value and its 95% confidence interval. We will use the *I*^2^ value and *P*-value to evaluate whether there is statistical heterogeneity. When *P*-value ≥ .1 and *I*^2^ value ≤ 50%, it means that there is no statistical heterogeneity between studies. We will use the fixed effect model. When *P*-value < .1 and *I*^2^ value > 50%, it means that there is statistical heterogeneity between studies, and then it is necessary to analyze the causes of heterogeneity, such as country, sex, age, course of disease, and other factors. If the heterogeneity is caused by these reasons, we will use subgroup analysis and sensitivity analysis as well as meta regression to further solve the problem. If there is still heterogeneity, we will choose the random effect model. If the above methods still can not solve the problem of heterogeneity, we will abandon meta-analysis and adopt descriptive analysis.

### 2.8 Ethics and dissemination

Since the data of this study are all from the literature and do not involve any personal privacy, there is no need for ethical approval. The results of this study will be published in a peer-reviewed journal or presented at a conference.

### 2.9 Assessment of the quality of evidence

The Grading of Recommendations Assessment, Development and Evaluation method will be used to assess the quality of the evidence obtained from this study.^[16]^

## 3 Discussion

Scapulohumeral periarthritis is mainly characterized by severe pain and limited movement of shoulder joint. It brings not only great physical pain to patients, but also great psychological pain such as anxiety, irritability, insomnia, and so on.^[3–8]^ A large number of literatures show that there are many methods of complementary and alternative therapies in the treatment of scapulohumeral periarthritis, each of which has its own unique advantages. The therapeutic effects are definite. For example: Acupuncture and cupping therapy can both stimulate the meridian and acupoints to regulate qi and activate blood stasis to relieve pain.^[17–19]^ Massage can relieve pain and shoulder spasm, relax adhesive tissues, restore balance, improve body metabolism, and eliminate aseptic inflammation.^[20]^ Chinese Herbal Medicine and moxibustion can relieve pain, reduce muscle tension, enhance vascular permeability and dilatation, promote blood circulation, promote the excretion of tissue metabolites, and promote the absorption of inflammatory factors and pain mediators.^[21]^ Exercise therapy can improve shoulder function, release adhesions and avoid muscle atrophy.^[22]^ Taichi can help patients to relax body and mind more deeply by providing dynamic stretching.^[23]^ Yoga can relieve shoulder pain and improve the limitation of shoulder movement.^[24]^ Nevertheless, there is a lack of systematic comparison of the efficacy and safety of different complementary and alternative therapies. Therefore, we will use the NMA method to compare the advantages and disadvantages of different supplementary and replacement therapies, so as to provide a basis for clinicians and patients with scapulohumeral periarthritis. However, due to that the data of this study are derived not from the original data, but from references, and the limitation of language, the results of the analysis will inevitably have some deviation. Thus, we will pay attention to the relevant progress in the future to conduct further research. Complementary and alternative therapy is effective in scapulohumeral periarthritis, and it is relatively simple for doctors and patients to operate with few side effects and good long-term effects. Therefore, through this study, we hope to provide evidence support and guidance for patients and clinicians to choose reasonable treatment for scapulohumeral periarthritis.

## Author contributions

**Conceptualization:** Meihan Gao.

**Data curation:** Meihan Gao, Haibo Cong.

**Formal analysis:** Meihan Gao.

**Funding acquisition:** Haibo Cong.

**Investigation:** Meihan Gao.

**Methodology:** Meihan Gao.

**Project administration:** Meihan Gao.

**Resources:** Meihan Gao, Chuancheng Li, Xiuyun Qin.

**Software:** Meihan Gao, Chuancheng Li, Xiuyun Qin, Hongqiang An, Zhenyuan Jiang.

**Supervision:** Meihan Gao.

**Validation:** Meihan Gao.

**Visualization:** Meihan Gao.

**Writing – original draft:** Meihan Gao, Chuancheng Li.

**Writing – review & editing:** Meihan Gao, Haibo Cong.
